# Darcy resistance flow of Sutterby nanofluid with microorganisms with applications of nano-biofuel cells

**DOI:** 10.1038/s41598-022-11528-7

**Published:** 2022-05-07

**Authors:** Abdulmajeed Aldabesh, A. Haredy, Kamel Al-Khaled, Sami Ullah Khan, Iskander Tlili

**Affiliations:** 1 Department of Mechanical Engineering, Faculty of Engineering, Albaha University, Al Bahah, 65527 Saudi Arabia; 2 Department of Architecture, Faculty of Engineering, Albaha University, Al Bahah, 65527 Saudi Arabia; 3 Department of Mathematics and Statistics, Jordan University of Science and Technology, P.O. Box 3030, Irbid, 22110 Jordan; 4 Department of Mathematics, COMSATS University Islamabad, Sahiwal, 57000 Pakistan; 5 Physics Department, College of Science Al-Zulfi, Majmaah University, Al-Majmaah, 11952 Saudi Arabia

**Keywords:** Energy science and technology, Engineering, Mathematics and computing

## Abstract

The objective of current research is to endorse the thermal aspect of Sutterby nanofluid containing the microorganisms due the stretched cylinder. The features of nonlinear thermal radiation, Darcy resistance and activation energy are also incorporated to inspect the thermal prospective. The problem is further extended with implementation of modified Fourier and Fick’s theories. The results are presented for the stretched cylinder and also for stationary plate. The numerical formulation for the problem is presented by following the shooting technique. The comparative numerical is performed to verify the computed simulations. The results convey that the presence of Darcy resistance parameter enhanced the velocity more effectively for stretched cylinder. A reduction in velocity due to Sutterby fluid parameter and buoyancy ratio parameter has been observed. Moreover, the temperature profile enhanced with larger sponginess parameter more effectively for stretching cylinder.

## Introduction

In today's growing world of technology, most engineers, scientists and researchers are focused on the analysis of nanoliquid due to their physical applications in the field of applied sciences. Nanofluids are known to be the accumulation of micro-shape solid objects in the convectional fluid. The accumulation of micro-shaped solid objects in the convection fluid is considered to be nanofluids. The enhancement in the heating capacity is necessary in different range of industrial and engineering mechanisms. The availability of increasing heat source is necessary to improve the optimal growth of industrial products. The communication and growing research in nanofluids predict novel thermal applications of such particle in the energy sector, power plants, chemical processes, engineering devices, thermal mechanisms etc. Choi^1^ explored the novel thermal aspect of nanofluids in primary research via experimental support. Alharbi^2^ studied flow of hybrid nanofluids with heat energy impacts. Uddinet al.^3^ scrutinized the radiative slip impact in nanofluid. Hassan et al.^4^ focused the thermal availably of hybrid nanofluid by assuming the shear thinning as a base liquid. Tlili et al.^5^ premeditated the thermal flow of Oldroyd-B nanofluid with isotropic slip impact. Khan et al.^6^ analyzed Darcy-Forchheimer flow in hybrid nanofluid. Haq et al.^7^ focused the improve thermal properties of nanofluid with suspension of Casson liquid. Xia et al.^8^ addressed the natural convective optimized analysis for Eyring-Powell nanofluid subject to microorganisms. The cross nanofluid flow with entropy generation assessment was utilized by Haq et al.^9^. In another reports, Haq et al.^10^ inspected the bioconvection applications for the nanofluid flow with controlled optimized phenomenon. Hussain et al.^11^ observed the carbon nanotube thermal outcomes with fluctuation of dynamic viscosity. The melting applications for hybrid nanofluid in addition of variable viscosity has been intended by Hussain et al.^12^. The analysis of Hussain et al.^13^ reports the improved heat transfer analysis for Jeffrey material with external heat source. Some more recent research on nanofluids is referred to refs.^14–17^.

The Sutterby nanofluid study is another topic of interest to enhance the thermal features of base fluids. Mir et al.^18^ addressed the relative improvement of heat transportation phenomenon by endorsing the Sutterby nanofluid in base liquid. The thermal research via interaction of Sutterby nanofluid with progressive thermal change was explored by Nawaz et al.^19^. Bilal et al.^20^ observed the role of magnetic force for Sutterby nanofluid flow. The influence of thermal radiation and the inclined magnetic field on the Sutterby fluid by focusing on the Cattaneo-Christov heat flux structure is examined by Sabir et al.^21^. Song et al.^22^ performed the Marangoni convection analysis for Sutterby nanofluid with melting and solutal constraints. Abbasi et al.^23^ endorsed the Sutterby nanoparticles properties in trapped channel.

The self-induced motile micro-organisms can increase the density of ordinary fluids in specific direction and, as a response a bioconvection phenomenon has evolved. Such nanoparticles motion is independent of microorganism movement and therefore the collective functionality of bioconvection and nanofluids seems to also be vital for microfluidics devices. Waqas et al.^24^ reported the bio-convective model for generalized viscoelastic nanofluid by performing the numerical simulations. Aziz et al.^25^ tackled a bidirectional bioconvection thermal nanofluid problem subject to accelerating space. Khan et al.^26^ discovered the theoretical continuation for couple stress nanofluid with consequences of activation energy and bioconvection phenomenon. Tong et al.^27^ explored the suspension of microorganisms subject to slip implementation for nanofluid. Li et al.^28^ reported the bio-convection applications for modified second grade fluid. Alwatban et al.^29^ expressed the physical onset of bioconvection phenomenon regarding the nanofluid flow.

Although a lot of research have been performed on the nanofluids, however, the thermal applications of Sutterby nanofluid subject to the bioconvection phenomenon with diverse flow features has not been performed yet. One this end, this research presents the thermal flow of Sutterby nanofluid containing the microorganisms due to moving cylinder. The navel aspects of this model are:

- The Darcy resistance flow of radiated Sutterby nanofluid with microorganisms due moving cylinder is presented.
- The Fourier and Fick’s modified expressions are used for examining the heat and mass transfer phenomenon.
- The novel thermal features like nonlinear thermal radiation and activation energy are also incorporated.
- The convective boundary conditions are utilized with motivations of enhancing the thermal transport of Sutterby nanofluid.
- The shooting technique for the formulated boundary value problem is implemented for obtained numerical simulations.
- The obtained simulations may present novel significances in bio-fuels, enzymes, thermal processes, energy systems, heat transfer devices etc.

## Flow model

**Figure 1:**
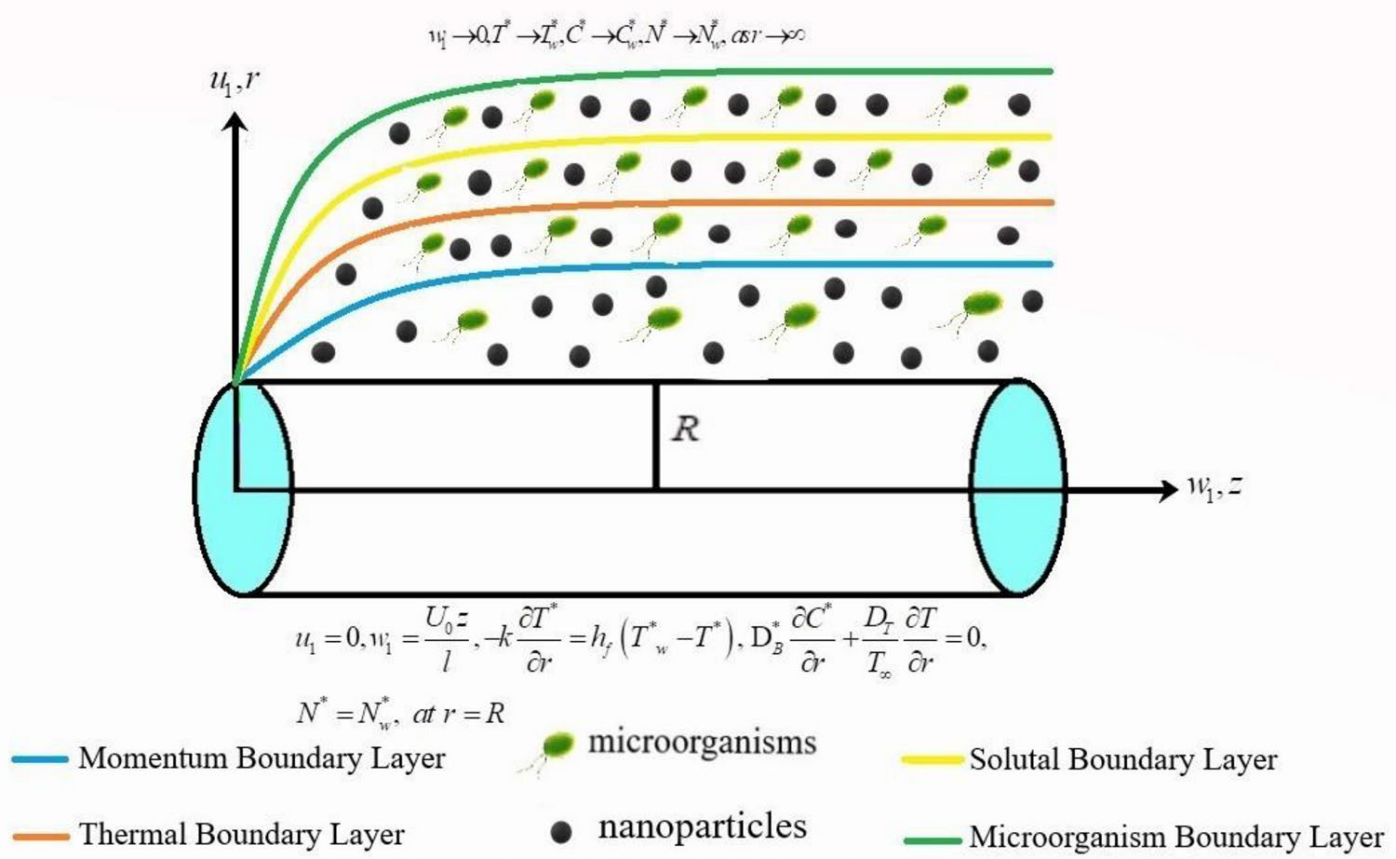
Physical view of flow.

The thermal transport of Sutterby nanofluid with consideration of suspension of microorganisms is taken into consideration. The stretched cylinder is assumed to originate the laminar flow. The velocity of moving cylinder is attributed to be $U_{w} \left( z \right) = U_{0} z/l.$ The normal aspect of magnetic force is also utilized^30^. The consequence of activation energy for solutal transport is discussed. A physical schematic of flow model is depicted in Fig. 1. Moreover $T_{w}^{*}$,$C_{w}^{*}$ and $N_{w}^{*}$ signifies wall surface temperature, concentration and microorganisms respectively. Here ambient temperature, concentration and microorganisms are symbolized by $T_{\infty }^{*}$, $C_{\infty }^{*}$ and $N_{\infty }^{*}$ correspondingly.

Under the above postulations, the leading governing boundary layer equations of flow are given as follows^18–20^:

1 $$ \frac{{\partial u_{1} }}{\partial r} + \frac{{u_{1} }}{r} + \frac{{\partial w_{1} }}{\partial z} = 0, $$

2 $$ \begin{gathered} u_{1} \frac{{\partial w_{1} }}{\partial r} + w_{1} \frac{{\partial w_{1} }}{\partial z} = \frac{{\nu_{a} }}{2}\frac{{\partial^{2} w_{1} }}{{\partial r^{2} }} + \frac{{\nu_{a} }}{2r}\frac{{\partial w_{1} }}{\partial r} - \frac{{\nu_{a} m^{*} \alpha^{2} }}{4}\left( {\frac{{\partial w_{1} }}{\partial r}} \right)^{2} \frac{{\partial^{2} w_{1} }}{{\partial r^{2} }} + \frac{{\sigma B_{o}^{2} }}{{\rho_{f} }}w_{1} \hfill \\ + \frac{{R_{z} }}{{\rho_{f} }} + \frac{1}{{\rho_{f} }}\left[ \begin{gathered} \left( {1 - C_{f} } \right)\rho_{f} \beta^{**} g*\left( {T - T_{\infty } } \right) - \left( {\rho_{p} - \rho_{f} } \right)g^{*} \left( {C - C_{\infty } } \right) \hfill \\ - \left( {N - N_{\infty } } \right)g^{*} \gamma \left( {\rho_{m} - \rho_{f} } \right) \hfill \\ \end{gathered} \right], \hfill \\ \end{gathered} $$

3 $$ \begin{gathered} \left[ {u_{1} \frac{{\partial T^{*} }}{\partial r} + w_{1} \frac{{\partial T^{*} }}{\partial z}} \right] + \lambda_{a} C_{H}^{*} = \frac{{K^{*} }}{{\left( {\rho_{f} C_{p} } \right)}}\left[ {\frac{{\partial^{2} T^{*} }}{{\partial r^{2} }} + \frac{1}{r}\frac{{\partial T^{*} }}{\partial r}} \right] \hfill \\ + \frac{\tau }{{\left( {\rho_{f} C_{p} } \right)}}\left[ {D_{B}^{*} \frac{{\partial C^{*} }}{\partial r}\frac{{\partial T^{*} }}{\partial r} + \frac{{D_{{T^{*} }}^{*} }}{{D_{B}^{*} }}\left( {\frac{{\partial T^{*} }}{\partial r}} \right)^{2} } \right] + \frac{1}{{\left( {\rho c} \right)_{f} }}\frac{1}{r}\frac{\partial }{\partial r}\left( {r\frac{{16\sigma^{*} }}{{3k^{*} }}T^{*3} \frac{{\partial T^{*} }}{\partial r}} \right), \hfill \\ \end{gathered} $$

Here,

4 $$ \begin{gathered} C_{H}^{*} = u_{1}^{2} \frac{{\partial^{2} T^{*} }}{{\partial r^{2} }} + w_{1}^{2} \frac{{\partial^{2} T^{*} }}{{\partial z^{2} }} + 2u_{1} w_{1} \frac{{\partial^{2} T^{*} }}{\partial r\partial z} + u_{1} \frac{{\partial u_{1} }}{\partial r}\frac{{\partial T^{*} }}{\partial z} + u_{1} \frac{{\partial w_{1} }}{\partial r}\frac{{\partial T^{*} }}{\partial z} + w_{1} \frac{{\partial u_{1} }}{\partial z}\frac{{\partial T^{*} }}{\partial r} \hfill \\ + w_{1} \frac{{\partial w_{1} }}{\partial r}\frac{{\partial T^{*} }}{\partial z} \hfill \\ \end{gathered} $$

5 $$ \begin{gathered} \left[ {u_{1} \frac{{\partial C^{*} }}{\partial r} + w_{1} \frac{{\partial C^{*} }}{\partial z}} \right] + \lambda_{a} C_{H}^{*} = D_{B}^{*} \left[ {\frac{{\partial^{2} C^{*} }}{{\partial r^{2} }} + \frac{1}{r}\frac{{\partial C^{*} }}{\partial r}} \right] + \frac{{D_{{T^{*} }}^{*} }}{{D_{B}^{*} }}\left[ {\frac{{\partial^{2} T^{*} }}{{\partial r^{2} }} + \frac{1}{r}\frac{{\partial T^{*} }}{\partial r}} \right] \hfill \\ - Kr^{2} \left( {C^{*} - C_{\infty }^{*} } \right)\left( {\frac{{T^{*} }}{{T_{\infty }^{*} }}} \right)^{n} \exp \left( {\frac{{ - E_{a} }}{{kT^{*} }}} \right), \hfill \\ \end{gathered} $$

where

6 $$ \begin{gathered} C_{H}^{*} = u_{1}^{2} \frac{{\partial^{2} C^{*} }}{{\partial r^{2} }} + w_{1}^{2} \frac{{\partial^{2} C^{*} }}{{\partial z^{2} }} + 2u_{1} w_{1} \frac{{\partial^{2} C^{*} }}{\partial r\partial z} + u_{1} \frac{{\partial u_{1} }}{\partial r}\frac{{\partial C^{*} }}{\partial z} + u_{1} \frac{{\partial w_{1} }}{\partial r}\frac{{\partial C^{*} }}{\partial z} \hfill \\ + w_{1} \frac{{\partial u_{1} }}{\partial z}\frac{{\partial C^{*} }}{\partial r} + w_{1} \frac{{\partial w_{1} }}{\partial r}\frac{{\partial C^{*} }}{\partial z} \hfill \\ \end{gathered} $$

7 $$ u_{1} \frac{{\partial N^{*} }}{\partial r} + w_{1} \frac{{\partial N^{*} }}{\partial z} + \left[ {\frac{\partial }{\partial r}\left( {N^{*} \frac{{\partial C^{*} }}{\partial r}} \right)} \right]\frac{{bW_{c} }}{{\left( {C_{w}^{*} - C_{\infty }^{*} } \right)}} = D_{m} \frac{\partial }{\partial r}\left( {\frac{{\partial N^{*} }}{\partial r}} \right), $$

with boundary conditions:

8 $$ \begin{gathered} u_{1} = 0,w_{1} = \frac{{U_{0} z}}{l}, - k\frac{{\partial T^{*} }}{\partial r} = h_{f} \left( {T^{*}_{w} - T^{*} } \right),\,\,D_{B}^{*} \frac{{\partial C^{*} }}{\partial r} + \frac{{D_{T} }}{{T_{\infty } }}\frac{\partial T}{{\partial r}} = 0,\,N^{*} = N_{w}^{*} ,\,\,\,at\,\,r = R\,, \hfill \\ w_{1} \to 0,T^{*} \to T_{w}^{*} ,C^{*} \to C_{w}^{*} ,N^{*} \to N_{w}^{*} ,\,\,as\,r \to \infty \, \hfill \\ \end{gathered} $$

In the above Eq. (2) Darcy resistance is defined as^31^:

9 $$ R = - \frac{\mu }{{2k^{*} }}\left[ {\frac{{\sinh^{ - 1} \left( {\alpha \gamma } \right)}}{\alpha \gamma }} \right]^{m} {\mathbf{V}}, $$

Introducing the following suitable similarities variables^18–20^:

10 $$ \begin{gathered} \zeta = \sqrt {\frac{{U_{0} }}{{\nu_{a} l}}} \left( {\frac{{r^{2} - R^{2} }}{2R}} \right),\,u_{1} = \sqrt {\frac{{\nu_{a} U_{0} }}{l}} \frac{R}{r}f\left( \zeta \right),\,w_{1} = - \frac{{U_{0} z}}{l}f^{\prime}\left( \zeta \right), \hfill \\ \theta \left( \zeta \right) = \frac{{T^{*} - T_{\infty }^{*} }}{{T_{w}^{*} - T_{\infty }^{*} }},\,\phi \left( \zeta \right) = \frac{{C^{*} - C_{\infty }^{*} }}{{C_{w}^{*} - C_{\infty }^{*} }},\chi \left( \zeta \right) = \frac{{N^{*} - N_{\infty }^{*} }}{{N_{w}^{*} - N_{\infty }^{*} }}. \hfill \\ \end{gathered} $$

After introducing the above appropriate transformation (10) in governing PDE’s, we acquire

11 $$ \begin{gathered} \left( {1 + 2\beta \zeta } \right)f^{\prime\prime\prime} + 2ff^{\prime\prime} - 2\left( {f^{\prime}} \right)^{2} - \frac{{\alpha_{1} }}{2}\left( {1 + 2\beta \zeta } \right)\left( {f^{\prime\prime}} \right)^{2} f^{\prime\prime\prime} + \beta f^{\prime\prime} - \left( {M + \frac{\alpha }{2}} \right)f^{\prime} + \frac{1}{12}\alpha_{2} f^{\prime}f^{\prime\prime} \hfill \\ + S^{*} \left[ {\theta - A_{1} \phi - A_{2} \,\chi } \right] = 0, \hfill \\ \end{gathered} $$

12 $$ \begin{gathered} \left[ {\left\{ {1 + Rd\left( {1 + \left( {\theta_{w} - 1} \right)\theta } \right)^{3} } \right\}\left( {1 + 2\beta \zeta } \right)\theta^{\prime}} \right]\theta^{\prime\prime} + 2\beta \theta^{\prime} + Prf\theta^{\prime} + Pr\left( {1 + 2\beta \zeta } \right)\left[ {Nb\theta^{\prime}\phi^{\prime} + Nt\left( {\theta^{\prime}} \right)^{2} } \right] \hfill \\ - Pr\lambda_{T} \left[ {ff^{\prime}\theta^{\prime} + f^{2} \theta^{\prime\prime}} \right] = 0, \hfill \\ \end{gathered} $$

13 $$ \begin{gathered} \left( {1 + 2\beta \zeta } \right)\phi^{\prime\prime} + 2\beta \phi^{\prime} + LePrf\phi^{\prime} + LePr\frac{Nt}{{Nb}}\left[ {\left( {1 + 2\beta \zeta } \right)\theta^{\prime\prime} + \beta \theta^{\prime}} \right] - LePr\lambda_{C} \left[ {ff^{\prime}\phi^{\prime} + f^{2} \phi^{\prime\prime}} \right] \hfill \\ - Le\Pr \sigma \left( {1 + \delta_{0} \theta } \right)^{n} \phi \exp \left( { - \frac{E}{{1 + \delta_{0} \theta }}} \right) = 0, \hfill \\ \end{gathered} $$

14 $$ \left( {1 + 2\beta \zeta } \right)\chi^{\prime\prime} + 2\beta \chi^{\prime} + Lb\chi^{\prime}f - Pe\left( {\phi^{\prime\prime}\left( {\chi + \varpi } \right) + \chi^{\prime}\phi^{\prime}} \right) = 0, $$

With

15 $$ \begin{gathered} f\left( \zeta \right) = 0,f^{\prime}\left( \zeta \right) = 1,\theta^{\prime} = - \gamma \left( {1 - \theta \left( \zeta \right)} \right),\, \hfill \\ Nb\phi^{\prime}\left( \zeta \right) + Nt\theta^{\prime}\left( \zeta \right)\,\,,\chi^{\prime}\left( \zeta \right) = 1\,\,\,at\,\zeta = 0, \hfill \\ f^{\prime} \to 0,\,\,\theta \to 0,\,\,\phi \to 0,\,\,\chi \to 0, \,\,\,as\,\,\zeta \to \infty \hfill \\ \end{gathered} $$

with dimensionless parameter:

16 $$ \begin{gathered} M = \frac{{l\sigma B_{0}^{2} }}{{\rho_{f} U_{0} }},S^{*} = \frac{{l^{2} \beta^{**} g^{*} \left( {1 - C_{\infty }^{*} } \right)\left( {T_{w}^{*} - T_{\infty }^{*} } \right)}}{{zU_{0}^{2} }},\beta = \frac{1}{R}\sqrt {\frac{{\nu_{a} l}}{{U_{0} }}} ,A_{1} = \frac{{\left( {C_{w}^{*} - C_{\infty }^{*} } \right)\left( {\rho_{p} - \rho_{f} } \right)}}{{\rho_{f} \beta^{**} \left( {1 - C_{w}^{*} } \right)\left( {T_{w}^{*} - T_{\infty }^{*} } \right)}}, \hfill \\ \alpha_{1} = \frac{{m^{*} \alpha^{2} U_{0}^{3} z^{2} }}{{l^{3} \nu_{a} }},A_{2} = \frac{{\gamma^{*} \left( {\rho_{m} - \rho_{f} } \right)\left( {N_{w}^{*} - N_{\infty }^{*} } \right)}}{{\rho_{f} \beta^{**} \left( {1 - C_{w}^{*} } \right)\left( {T_{w}^{*} - T_{\infty }^{*} } \right)}},\alpha_{2} = \frac{{m^{*} \alpha^{2} U_{0}^{2} z^{2} }}{{l^{2} k^{*} }},\alpha = \frac{{\nu_{a} l}}{{k^{*} U_{0} }},\lambda_{T} = \frac{{\lambda_{a} U_{0} }}{l}, \hfill \\ Pr = \frac{{\nu_{a} }}{\alpha },Nt = \left( {\frac{{\tau D^{*}_{{T^{*} }} \left( {T_{w}^{*} - T_{\infty }^{*} } \right)}}{{\nu_{a} T_{\infty }^{*} }}} \right),Le = \frac{\alpha }{{D_{B}^{*} }},Rd = \frac{{16\sigma^{*} T_{\infty }^{3} }}{{3kk^{*} }},\theta_{w} = \frac{{T_{w}^{*} }}{{T_{\infty }^{*} }},\sigma = \frac{{lKr^{2} }}{{U_{0} }},E = \frac{{E_{a} }}{{kT_{\infty } }}, \hfill \\ Nb = \left( {\frac{{\tau D_{B}^{*} \left( {C_{w}^{*} - C_{\infty }^{*} } \right)}}{{\nu_{a} }}} \right),\lambda_{C} = \frac{{\lambda_{b} U_{0} }}{l},\delta_{0} = \frac{{T_{w}^{*} - T_{\infty }^{*} }}{{T_{\infty }^{*} }},Lb = \frac{{\nu_{a} }}{{D_{m} }},Pe = \frac{{bW_{c} }}{{D_{m} }},\varpi = \frac{{N_{\infty }^{*} }}{{N_{w}^{*} - N_{\infty }^{*} }}, \hfill \\ \gamma = \frac{{h_{f} }}{k}\sqrt {\frac{{\nu_{a} l}}{{U_{0} }}.} \hfill \\ \end{gathered} $$

Physical quantities of interest are

17 $$ Nu_{z} = \frac{{zq_{m} }}{{k\left( {T_{w}^{*} - T_{\infty } } \right)}},Sh_{z} = \frac{{zj_{m} }}{{D_{B}^{*} \left( {C_{w}^{*} - C_{\infty }^{*} } \right)}},Sn_{z} = \frac{{zj_{m} }}{{D_{m}^{*} \left( {N_{w}^{*} - N_{\infty }^{*} } \right)}}. $$

$q_{m}$ the local heat flux,$j_{m}$ for local mass flux and $j_{n}$ microorganisms flux, which are addressed as

18 $$ q_{m} = - k\left( {\frac{\partial T}{{\partial r}}} \right)_{r = R} - ,j_{m} = - D_{B} \left( {\frac{\partial C}{{\partial r}}} \right)_{r = R} ,j_{n} = - D_{m} \left( {\frac{\partial N}{{\partial r}}} \right)_{r = R} $$

In the dimensionless forms are

19 $$ Nu_{z} {\text{Re}}_{z}^{{ - \frac{1}{2}}} = - \left( {1 + \frac{4}{3}R_{d} \left( {1 + \left( {\theta_{w} - 1} \right)\theta \left( 0 \right)} \right)} \right)^{3} \theta^{\prime}\left( 0 \right),Sh_{z} {\text{Re}}_{z}^{{ - \frac{1}{2}}} = - \phi^{\prime}\left( 0 \right),Sn_{z} {\text{Re}}_{z}^{{ - \frac{1}{2}}} = - \chi^{\prime}\left( 0 \right) $$

Here the local Reynolds number is symbolized by ${\text{Re}}_{z} = \frac{{U_{0} z}}{{\nu_{a} }}$.

## Numerical procedure

The coupled governing odrinary differential Eqs. (11–14) with boundary restrictions (15) is higly nonlinear in nature. The numerical solutions of these system is very diffecullt. There for obtain numerical solutions of model we employ shooting technique via Matlab tool bvp4c. initially, the higher-order ODE’s are converted into first order system, by implementing following procedure

20 $$ \left. \begin{gathered} f = t_{1} ,f^{\prime} = t_{2} ,f^{\prime\prime} = t_{3} ,f^{\prime\prime\prime} = t^{\prime}_{3} ,,\theta = t_{4} ,\theta^{\prime} = t_{5} ,\theta^{\prime\prime} = t^{\prime}_{5} ,\phi = t_{6} , \hfill \\ \phi^{\prime} = t_{7} ,\phi^{\prime\prime} = t^{\prime}_{7} ,\chi = t_{8} ,\chi^{\prime} = t_{9} ,\chi^{\prime\prime} = t^{\prime}_{9} \hfill \\ \end{gathered} \right\}, $$

21 $$ t^{\prime}_{3} = \frac{{ - 2tt_{3} + 2t_{2}^{2} - \beta t_{3} + \left( {M + \frac{\alpha }{2}} \right)t_{2} - \frac{1}{12}\alpha_{2} t_{2} t_{3} - S^{*} \left[ {t_{4} - A_{1} t_{6} - A_{2} t_{8} } \right]}}{{\left( {1 - \frac{{\alpha_{1} }}{2}t_{3}^{2} } \right)\left( {1 + 2\beta \zeta } \right)}}, $$

22 $$ t^{\prime}_{5} = \frac{{ - 2\beta t_{5} - Pr\,tt_{5} - Pr\left( {1 + 2\beta \zeta } \right)\left[ {Nbt_{5} t_{7} + Nt\,t_{5}^{2} } \right] + Pr\lambda_{T} \left[ {tt_{2} t_{5} } \right]}}{{\left( {\left[ {\left\{ {1 + Rd\left( {1 + \left( {\theta_{w} - 1} \right)t_{4} } \right)^{3} } \right\}\left( {1 + 2\beta \zeta } \right)t_{5} } \right] - Pr\lambda_{T} t^{2} } \right)}}, $$

23 $$ t^{\prime}_{7} = \frac{\begin{gathered} - 2\beta t_{7} - LePrtt_{7} - LePr\frac{Nt}{{Nb}}\left[ {\left( {1 + 2\beta \zeta } \right)t^{\prime}_{5} + \beta t_{5} } \right] + LePr\lambda_{C} \left[ {tt_{1} t_{7} } \right] \hfill \\ + LePr\sigma \left( {1 + \delta_{0} t_{4} } \right)^{n} t_{6} \exp \left( { - \frac{E}{{1 + \delta_{0} t_{4} }}} \right) \hfill \\ \end{gathered} }{{\left( {\left( {1 + 2\beta \zeta } \right) - LePr\lambda_{C} t^{2} } \right)}}, $$

24 $$ t^{\prime}_{9} = \frac{{ - 2\beta t_{9} - Lbt_{9} t + Pe\left( {t^{\prime}_{7} \left( {t_{8} + \varpi } \right) + t_{9} t_{7} } \right)}}{{\left( {1 + 2\beta \zeta } \right)}}, $$

25 $$ \begin{gathered} t\left( \zeta \right) = 0,t_{2} \left( \zeta \right) = 1,t_{5} = - \gamma \left( {1 - t_{4} \left( \zeta \right)} \right),\, \hfill \\ Nbt_{7} \left( \zeta \right) + Nt\,t_{5} \left( \zeta \right)\,\,,\,t_{9} \left( \zeta \right) = 1\,\,\,at\,\zeta = 0, \hfill \\ t_{2} \to 0,\,\,t_{4} \to 0,\,t_{6} \to 0,t_{8} \to 0, \,\,\,as\,\,\zeta \to \infty \hfill \\ \end{gathered} $$

## Validation of results

The solution verification and validity has been checked in Table 1 with comparison the numerical with investigation of Fathizadeh et al.^32^ and Fang et al.^33^. A fine accuracy of obtained results is noted with these studies.

**Table 1:** The comparative analysis when $\beta = S^{ * } = A_{1} = A_{2} = \alpha = 0.$

$M$	Fathizadeh et al.^32^			Fang et al.^33^	Present results
	HPM	MHPM	Exact solution		
0	1	1	1	1	1.0000
1.0	− 1.4142	− 1.4142	− 1.4142	− 1.4142	− 1.4145

## Results and discussion

**Figure 2:**
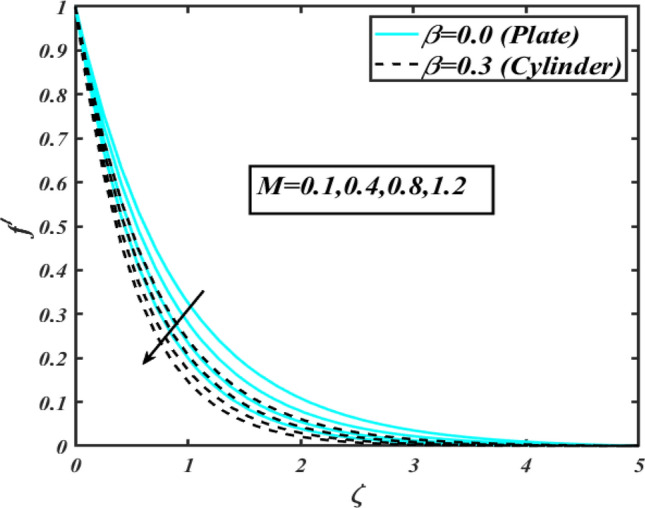
Profile of $f^{\prime}$ for $M$.

**Figure 3:**
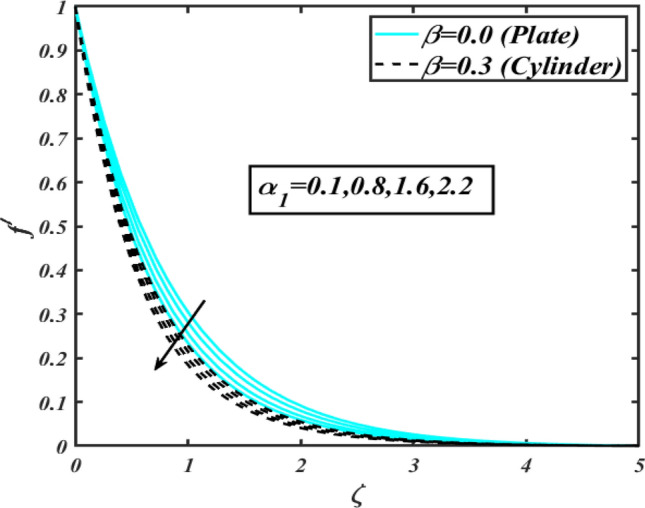
Profile of $f^{\prime}$ for $\alpha_{1}$.

**Figure 4:**
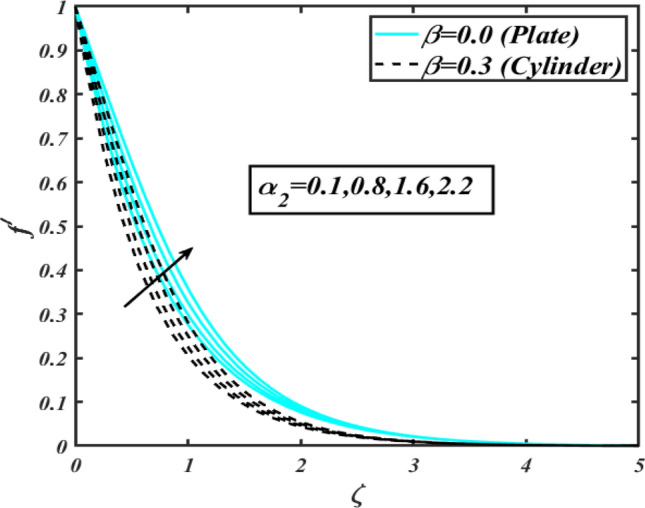
Profile of $f^{\prime}$ for $\alpha_{2}$.

**Figure 5:**
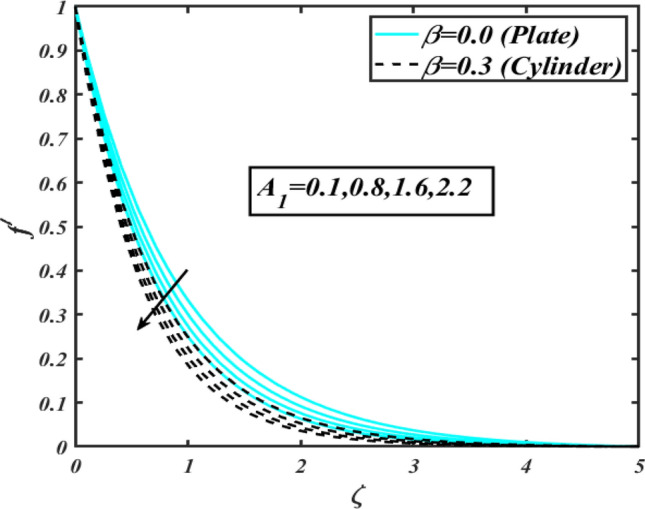
Profile of $f^{\prime}$ for $A_{1}$.

**Figure 6:**
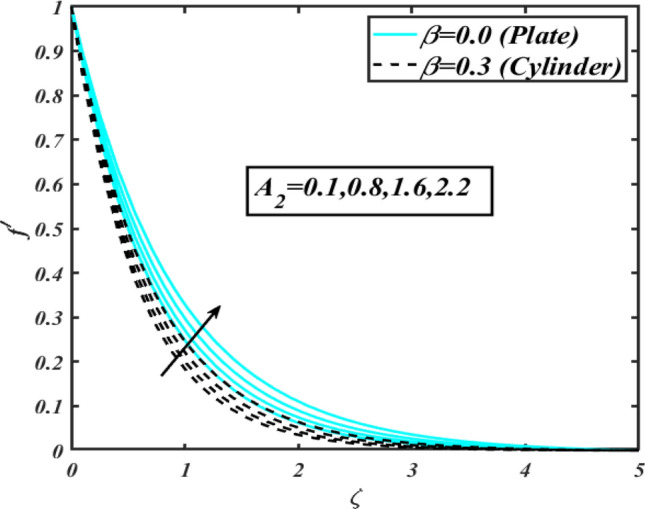
Profile of $f^{\prime}$ for $A_{2}$.

**Figure 7:**
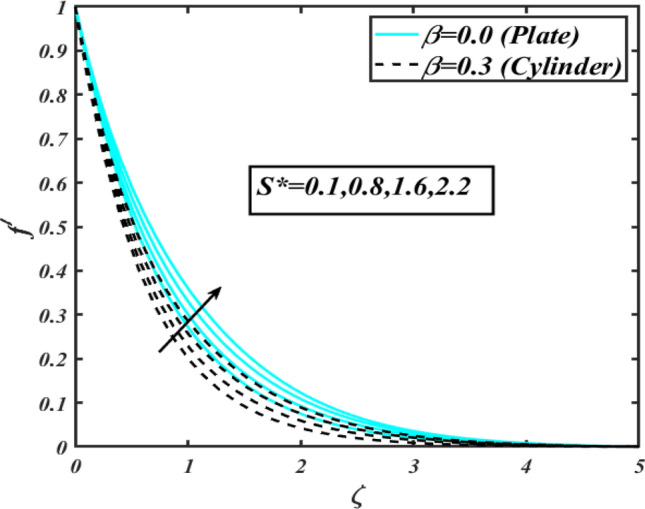
Profile of $f^{\prime}$ for $S^{*}$.

**Figure 8:**
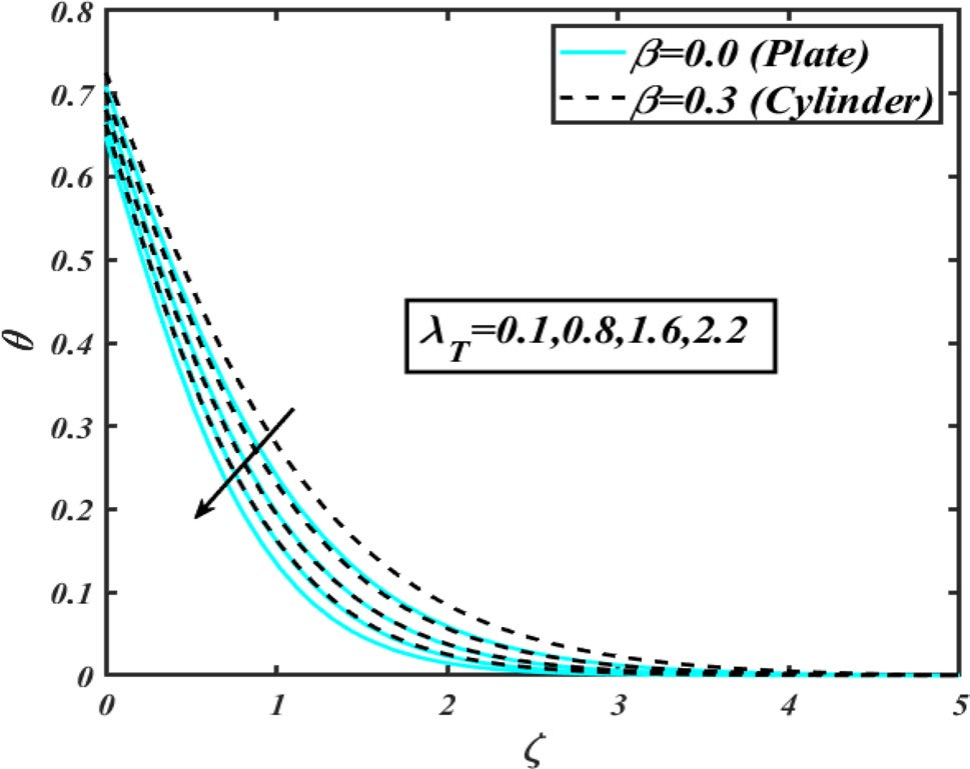
Profile of $\theta$ for $\lambda_{T}$.

**Figure 9:**
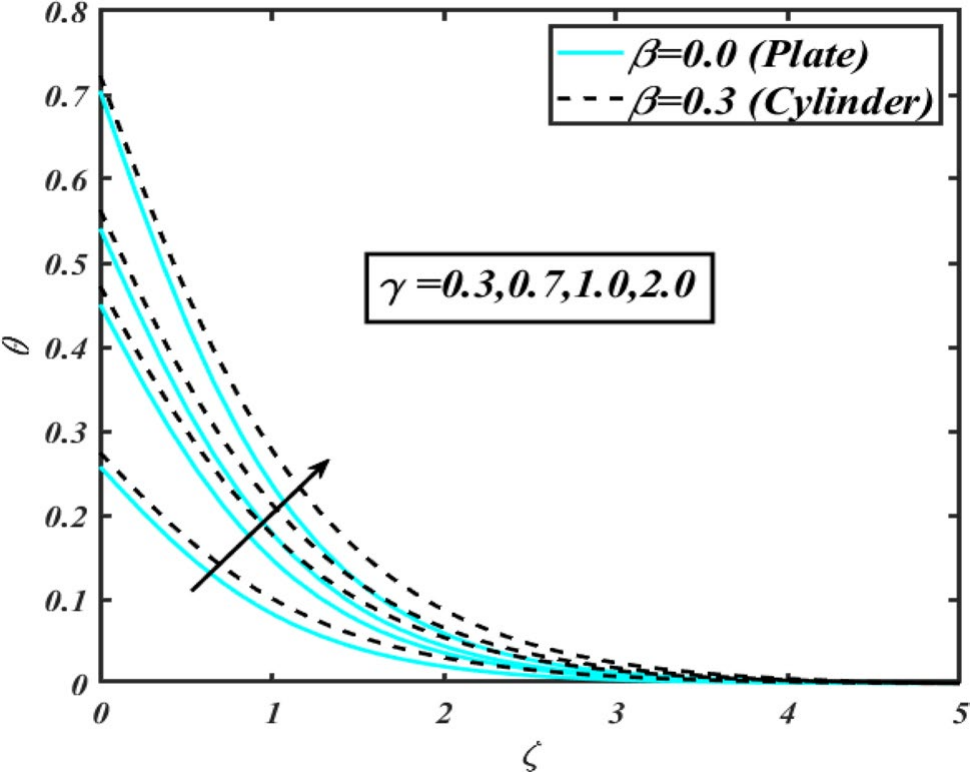
Profile of $\theta$ for $\gamma$.

**Figure 10:**
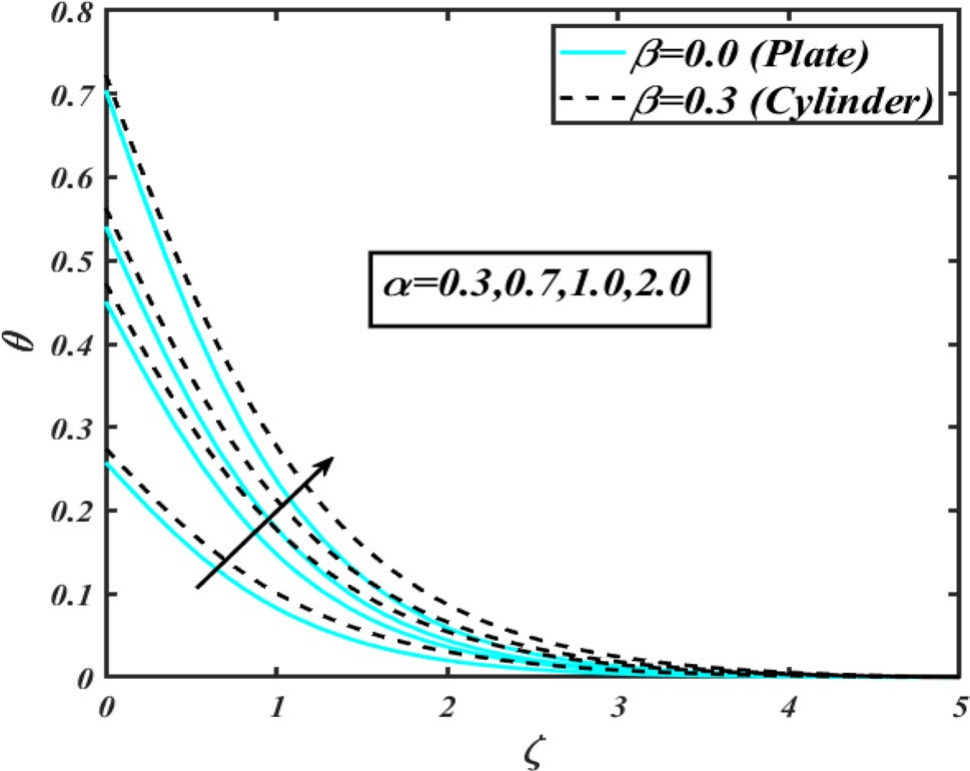
Profile of $\theta$ for $\alpha$.

**Figure 11:**
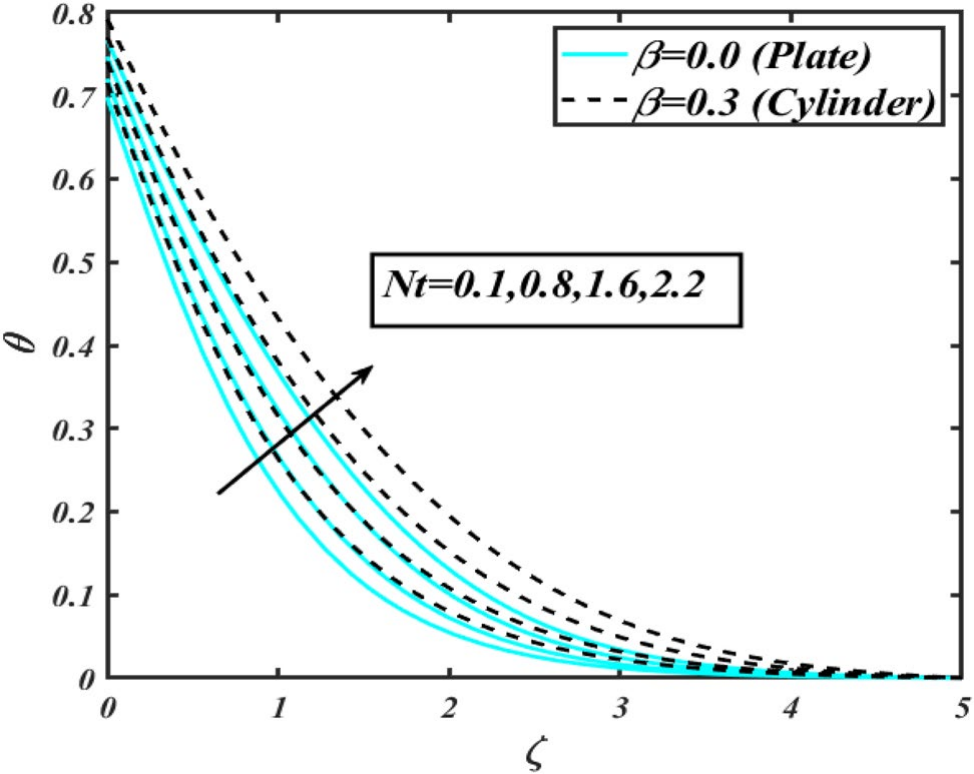
Profile of $\theta$ for $Nt$.

**Figure 12:**
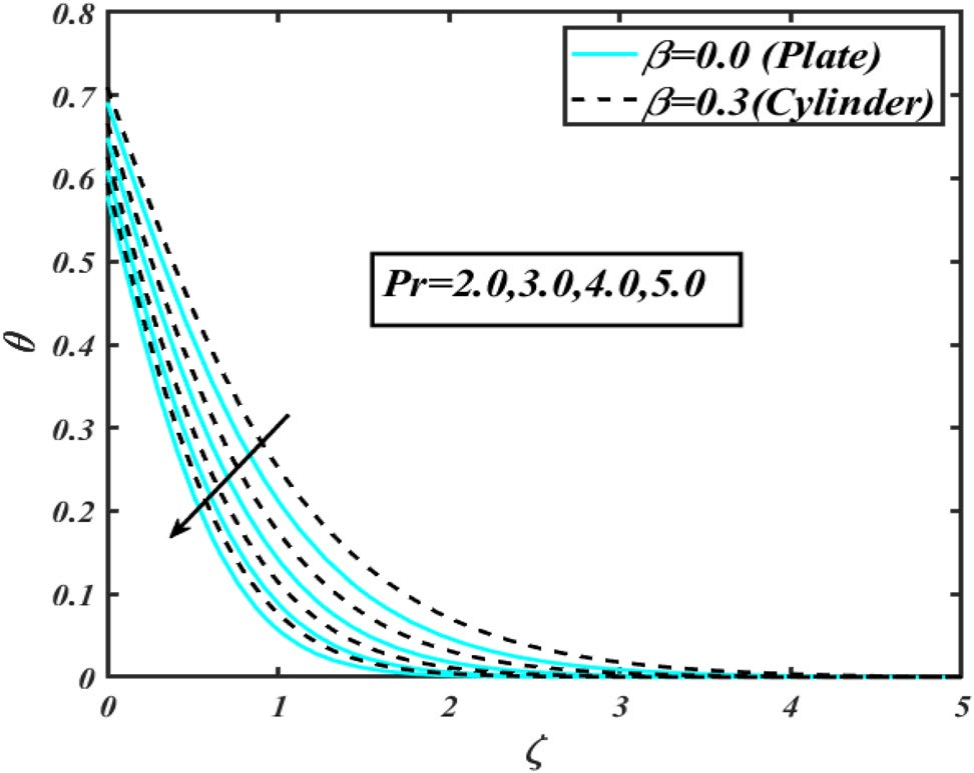
Profile of $\theta$ for $Pr$.

**Figure 13:**
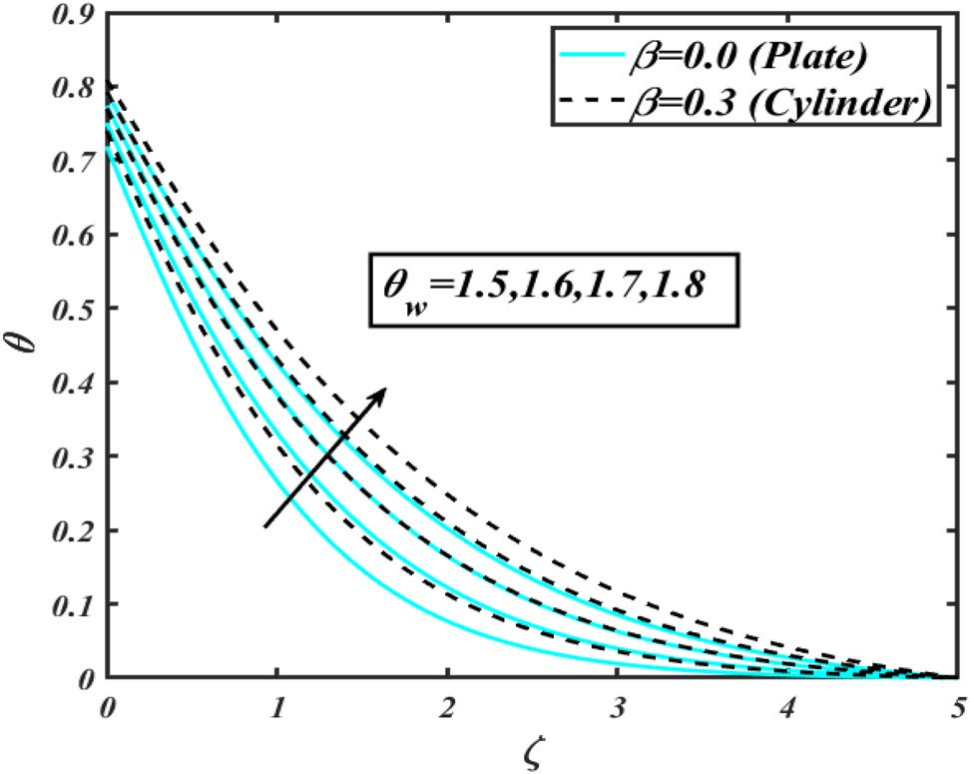
Profile of $\theta$ for $\theta_{w}$.

**Figure 14:**
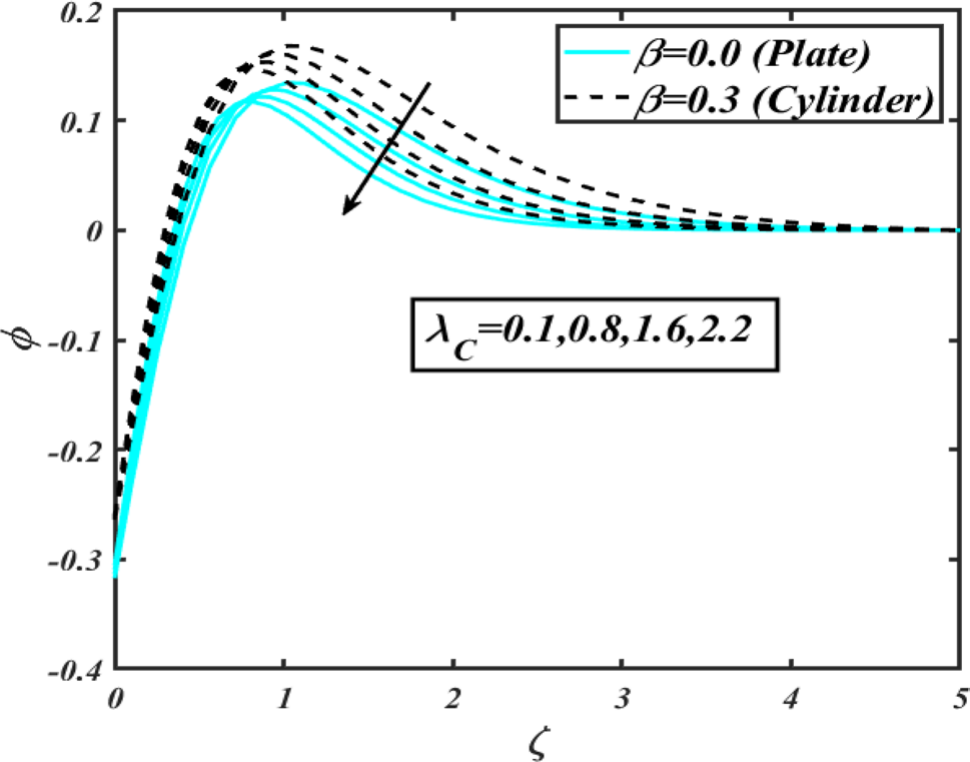
Profile of $\phi$ for $\lambda_{C}$.

**Figure 15:**
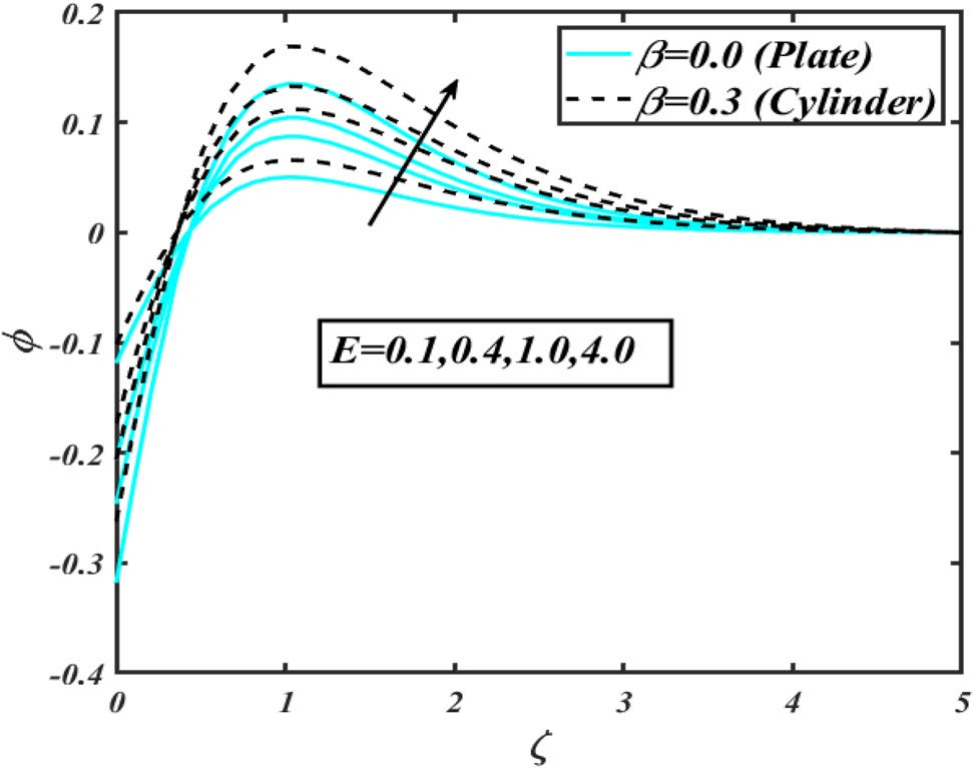
Profile of $\phi$ for $E$.

**Figure 16:**
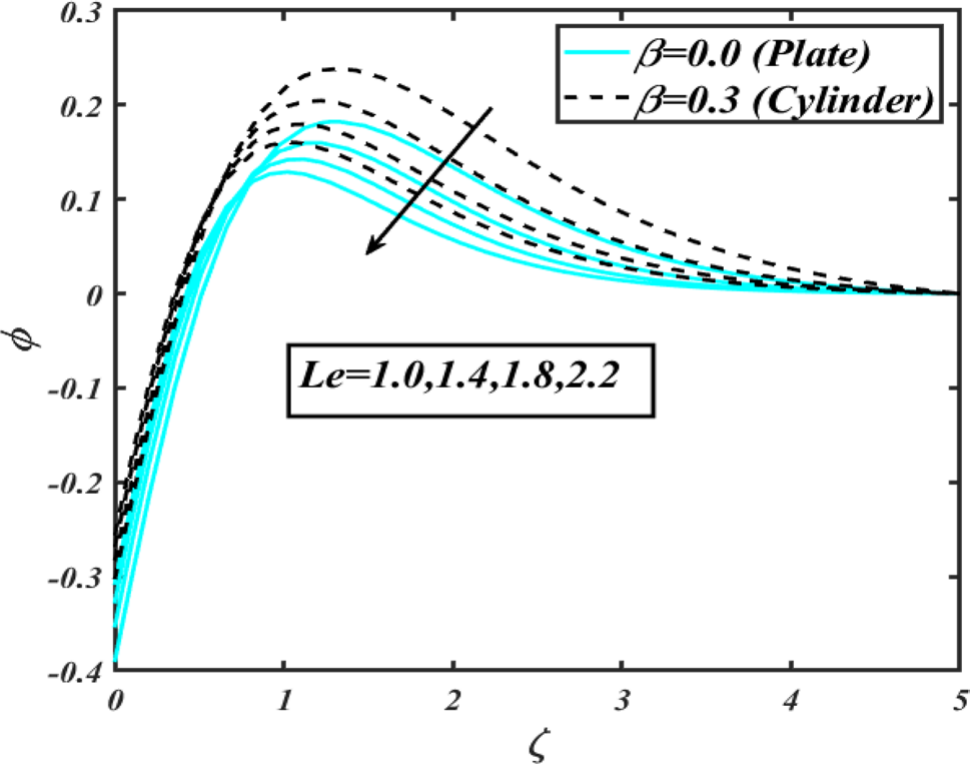
Profile of $\phi$ for $Le$.

**Figure 17:**
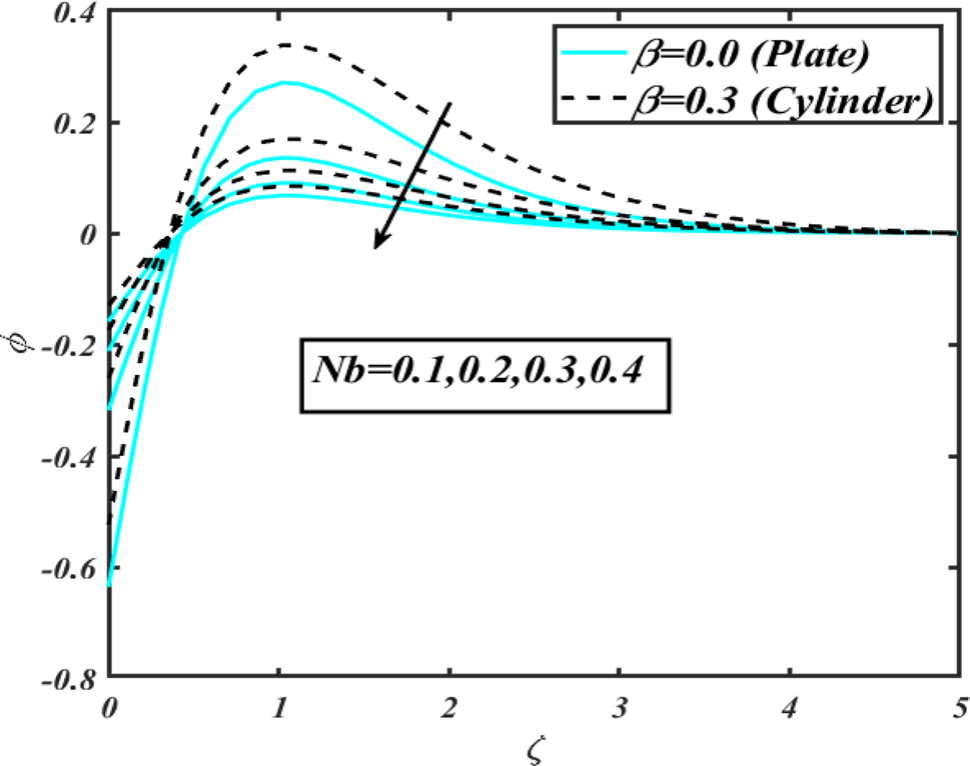
Profile of $\phi$ for $Nb$.

**Figure 18:**
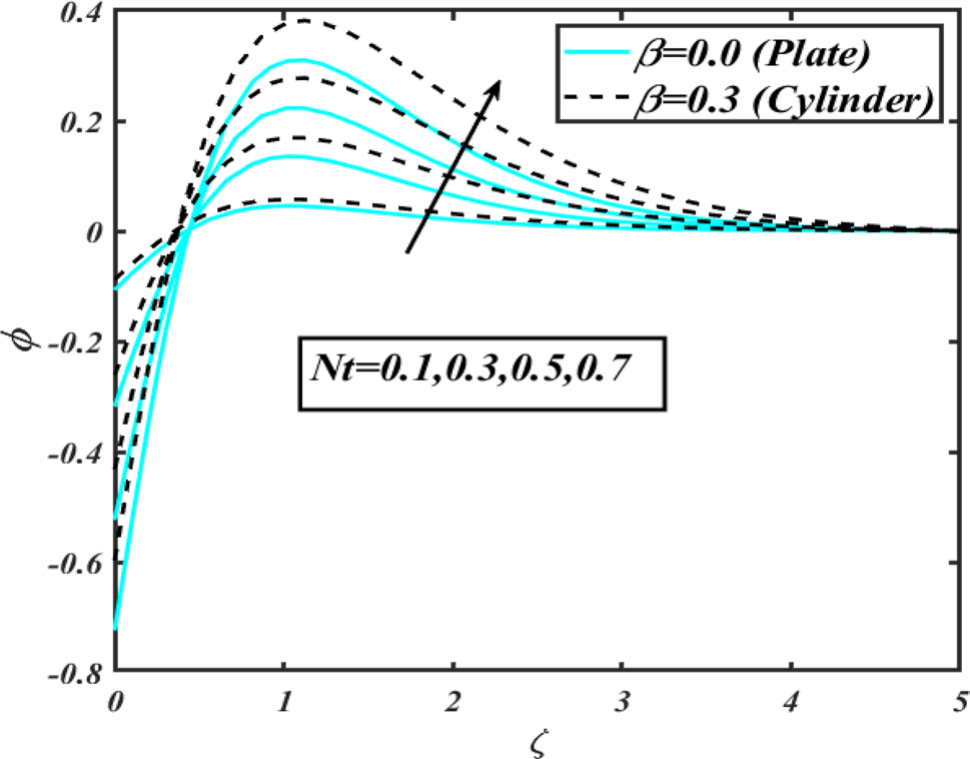
Profile of $\phi$ for $Nt$.

**Figure 19:**
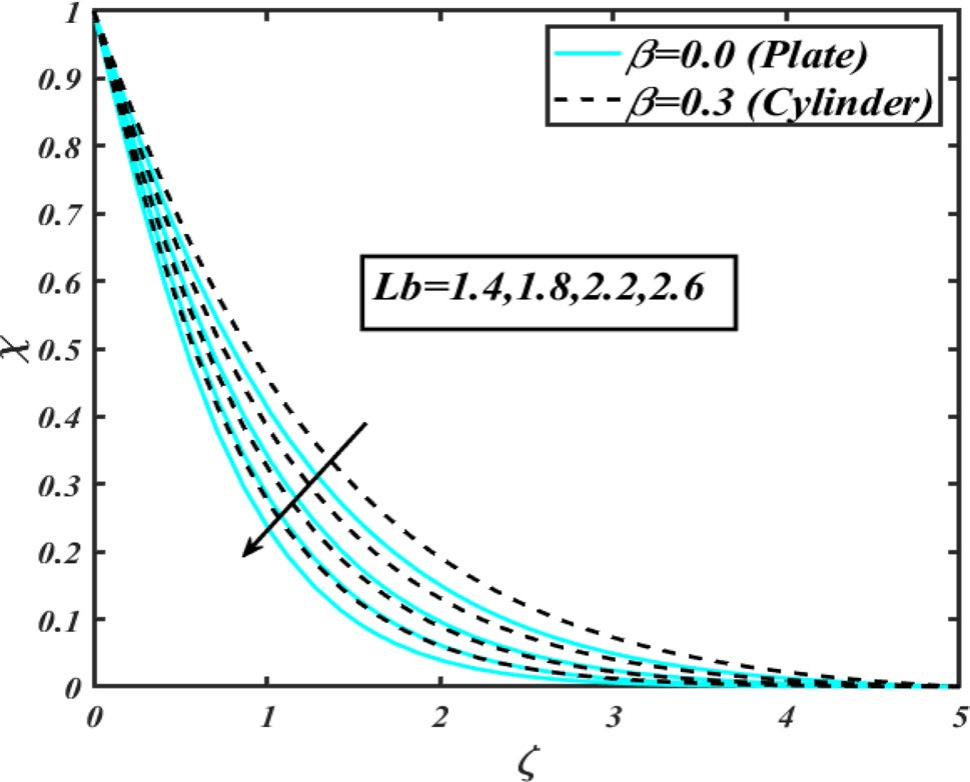
Profile of $\chi$ for $Lb$.

**Figure 20:**
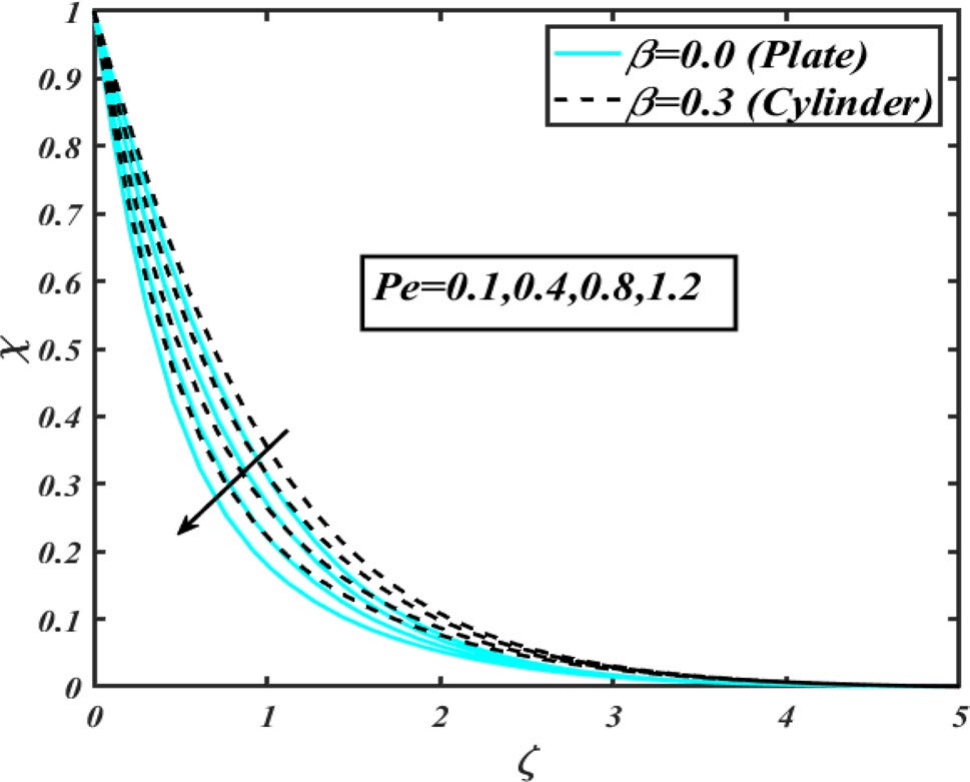
Profile of $\chi$ for $Pe$.

This section communicates the physical aspect of Sutterby nanofluid in view of flow parameters. The comparative analysis is performed for flow due to plate $\left( {\beta = 0.0} \right)$ and cylinder $\left( {\beta = 0.3} \right).$ Figure 2 is drawn to estimate the consequence of Hartmann number $M$ on flow velocity $f^{\prime}$. The interaction of magnetic force reports a declining change in velocity due to presence of Lorentz force. Moreover, the declining change in velocity is more progressive for plate as compared to stretched cylinder. Figure 3 characterizes the impact of Sutterby fluid parameter $\alpha_{1}$ on velocity $f^{\prime}$. The velocity $f^{\prime}$ dwindles for increasing change in Sutterby fluid parameter $\alpha_{1}$. The outcomes of $f^{\prime}$ against Darcy resistance parameter $\alpha_{2}$ is delineated via Fig. 4. An increasing change in velocity enhanced the $f^{\prime}$ for Darcy resistance parameter $\alpha_{2}$. Figure 5 is portrayed to understand the impact of buoyancy ratio parameter $A_{1}$ on $f^{\prime}$ It is analyzed that $f^{\prime}$ reduces for increasing variation of $A_{1}$ for both plate $\left( {\beta = 0.0} \right)$ and stretched cylinder $\left( {\beta = 0.3} \right)$. Physically, the buoyancy forces play novel contribution to reduce the velocity rate effectively. Figure 6 is plotted to investigate the insight of bioconvection Rayleigh factor $A_{2}$ on $f^{\prime}$. The velocity field $f^{\prime}$ exaggerates for larger bioconvection Rayleigh number $A_{2}$. Figure 7 is illustrating the deviation of mixed convection parameter $S^{*}$ on $f^{\prime}$ for both plate and cylinder. The depicted change in $f^{\prime}$ show an increasing fluctuation by raise in estimation of mixed convection parameter $S^{*}$. Physically, the mixed convection constant explores the ratio between buoyancy to viscous force. The increasing contributions of buoyancy forces results an increment in the velocity. The inspiration of thermal relaxation time parameter $\lambda_{T}$ against temperature profile $\theta$ is illustrated in Fig. 8. The temperature profile $\theta$ reduces for larger number of thermal relaxation parameter $\lambda_{T}$. Figure 9 manifests the effect of Biot number $\gamma$ on $\theta$. It is seen that $\theta$ increased against larger values of Biot number $\gamma$. Physically, the Biot number present the heat transfer coefficient which enhanced the temperature profile. Moreover, the rate of heat transfer is relatively more growing for stretched cylinder as compared to plate. To consequence of sponginess parameter $\alpha$ on $\theta$, Fig. 10 is pictured. It is predicted that $\theta$ upsurges for larger values of sponginess parameter $\alpha$. Fig. 11 is inserted to envision the effect of thermophoresis parameter $Nt$ on thermal field $\theta$ of fluid. The thermophoresis phenomenon is based on the collection of nanoparticles which migrated to the cooler surface because of temperature gradient. This fluctuation in temperature due to thermophoresis phenomenon increase the temperature profile. Figure 12 reports the inspiration of Prandtl number $Pr$ on $\theta$. The lower temperature changes have been noted due $Pr$ is noted. Physically, the increasing outcomes in Prandtl number declined the thermal diffusivity due to which $\theta$ declined. Figure 13 impacted the change in $\theta$ due to temperature ratio parameter $\theta_{w}$. It is analyzed that $\theta$ shows an enlarging trend for growing values of temperature ratio parameter $\theta_{w}$. The effect of solutal relaxation time parameter $\lambda_{C}$ against concentration field $\phi$ is deliberated in Fig. 14. The concentration profile $\phi$ reduces for $\lambda_{C}$. The features of activation energy parameter $E$ on $\phi$ is scrutinized in Fig. 15. It is perceived that $\phi$ enhanced by raising the numbers of activation energy parameter $E$. The activation energy determines the minimum energy supply to start the reaction phenomenon. The presence of activation energy enhanced the concentration change more effectively. The physical aspect of Lewis number $Le$ on $\phi$ is examined via Fig. 16. The concentration $\phi$ reduces by augmenting the values of Lewis number $Le$. Physically, this reduction in concentration is due to low mass diffusivity of nanoparticles associated to the higher values of Lewis number. The physical outcomes of Brownian motion parameter $Nb$ against $\phi$ of is considered in Fig. 17. The diminishes change in $\phi$ against larger $Nb$ is noticed. The characteristics of thermophoresis parameter $Nt$ against $\phi$ is characterized in Fig. 18. The concentration rate $\phi$ is dwindles with larger thermophoresis parameter $Nt$. Figure 19 is presented to estimate the variation of bioconvection Lewis number $Lb$ on microorganism field $\chi$ for plate $\left( {\beta = 0.0} \right)$ and cylinder $\left( {\beta = 0.3} \right).$ It is analyzed that microorganism field $\chi$ declines with enlarge numbers of $Lb$. The physical features of Peclet number $Pe$ on $\chi$ disclosed in Fig. 20. The lower microorganism rate for higher $Pe$ is observed. The lower microorganism profile with higher Peclet number is owing to low motile diffusivity.

The numerical data is achieved for inspecting the heat transfer rate, mass transfer pattern, motile density and wall shear forces in tables (2, 3, 4, 5). Table 2 suggests that the wall shear rate enhance with Hartmann number while it reduces for mixed convection parameter. The numerical observations are relatively growing for stretched cylinder. The numerical outcomes listed in Table 3 explores the Nusselt number variation when different numerical values are assigned to parameters. The decreasing numerical data is achieved for thermophoresis constant while increasing observations are predicted against thermal relaxation constant. Table 4 notify that the Sherwood number numerical variation is larger for Lewis number and concentration relaxation number. From Table 5, the enhanced numerical data is results against the Peclet number.

**Table 2:** Outcomes of $- f^{\prime\prime}\left( 0 \right)$ versus $M$, $S^{*}$, $A_{1}$, $A_{2}$, $\alpha_{1}$ and $\alpha_{2}$.

Parameters						$- f^{\prime\prime}\left( 0 \right)$	
$M$	$S^{*}$	$A_{1}$	$A_{2}$	$\alpha_{1}$	$\alpha_{2}$	$\beta = 0.0$	$\beta = 0.3$
0.1	0.2	0.1	0.1	0.1	1.0	1.0193	1.3106
0.8						1.3058	1.5981
1.6						1.5705	1.8620
0.5	0.1	0.1	0.1	0.1	1.0	1.1914	1.3598
	1.0					1.1887	1.3469
	2.0					0.1825	1.3376
0.5	0.2	0.2	0.1	0.1	1.0	1.1911	1.4835
		0.8				1.1892	1.4819
		1.6				1.1866	1.4799
0.5	0.2	0.1	0.2	0.1	1.0	1.1922	1.4846
			0.8			1.1969	1.4895
			1.6			1.2030	1.4961
0.5	0.2	0.1	0.1	0.1	1.0	1.1923	1.4787
				0.6		1.1975	1.4867
				1.2		1.2042	1.4939
0.5	0.2	0.1	0.1	0.1	0.1	0.1936	1.4809
					0.6	0.1865	1.4650
					1.2	0.1821	1.4318

**Table 3:** Outcomes of $- f^{\prime\prime}\left( 0 \right)$ versus $M$, $S^{*}$, $A_{1}$, $A_{2}$, $Nt$, $Nb$, $Pr$, $Rd$, $\lambda_{T}$ and $Le$.

Parameters										$- \theta^{\prime}\left( 0 \right)$	
$M$	$S^{*}$	$A_{2}$	$A_{1}$	$Nt$	$Nb$	$Pr$	$Rd$	$\lambda_{T}$	$Le$	$\beta = 0.0$	$\beta = 0.3$
0.1	0.2	0.1	0.1	0.3	0.2	2	0.8	0.3	2.0	0.6059	0.5734
0.8										0.5726	0.5399
1.6										0.5423	0.5423
0.5	0.1	0.1	0.1	0.3	0.2	2	0.8	0.3	2.0	0.5724	0.5480
	1.6									0.5836	0.5317
	2.0									0.5980	0.5058
0.5	0.2	0.2	0.1	0.3	0.2	2	0.8	0.3	2.0	0.5858	0.5528
		0.8								0.5849	0.5517
		1.6								0.5836	0.5503
0.5	0.2	0.1	0.2	0.3	0.2	2	0.8	0.3	2.0	0.5859	0.5530
			0.8							0.5960	0.5529
			1.6							0.5861	0.5528
0.5	0.2	0.1	0.1	0.1	0.2	2	0.8	0.3	2.0	0.5954	0.5643
				0.6						0.5714	0.5359
				1.2						0.5411	0.5008
0.5	0.2	0.1	0.1	0.3	0.1	2	0.8	0.3	2.0	0.5952	0.5560
					0.6					0.5711	0.5423
					1.2					0.5352	0.5081
0.5	0.2	0.1	0.1	0.3	0.2	1.2	0.8	0.3	2.0	0.4603	0.4295
						2.2				0.6109	0.5782
						3.2				0.7109	0.6802
0.5	0.2	0.1	0.1	0.3	0.2	1.2	0.1	0.3	2.0	0.5767	0.5432
							0.6			0.5841	0.5510
							1.2			0.5885	0.5559
0.5	0.2	0.1	0.1	0.3	0.2	1.2	0.8	0.1	2.0	0.5811	0.5412
								0.6		0.5865	0.5532
								1.2		0.5889	0.5537
0.5	0.2	0.1	0.1	0.3	0.2	1.2	0.8	0.3	1.0	0.5874	0.5545
									2.0	0.5816	0.5487
									3.0	0.5781	0.5453

**Table 4:** Outcomes of $- f^{\prime\prime}\left( 0 \right)$ versus $M$, $S^{*}$, $A_{1}$, $A_{2}$, $Nt$, $Nb$, $Pr$, $\lambda_{C}$ and $Le.$

Parameters									$- \phi^{\prime}\left( 0 \right)$	
$M$	$S^{*}$	$A_{2}$	$A_{1}$	$Nt$	$Nb$	$Pr$	$\lambda_{C}$	$Le$	$\beta = 0.0$	$\beta = 0.3$
0.1	0.2	0.1	0.1	0.3	0.2	2	0.3	2.0	0.9089	0.8601
0.8									0.8589	0.8098
1.6									0.8135	0.7663
0.5	0.1	0.1	0.1	0.3	0.2	2	0.3	2.0	0.8642	0.8407
	1.6								0.8763	0.8371
	2.0								0.8860	0.8185
0.5	0.2	0.2	0.1	0.3	0.2	2	0.3	2.0	0.8787	0.8293
		0.8							0.8773	0.8276
		1.6							0.8754	0.8254
0.5	0.2	0.1	0.2	0.3	0.2	2	0.3	2.0	0.8789	0.8295
			0.8						0.8790	0.8294
			1.6						0.8791	0.8293
0.5	0.2	0.1	0.1	0.1	0.2	2	0.3	2.0	0.2977	0.2821
				0.6					1.7141	1.6076
				1.2					3.2467	3.0050
0.5	0.2	0.1	0.1	0.3	0.1	2	0.3	2.0	1.8632	1.6590
					0.6				0.8512	0.2767
					1.2				0.8489	0.1383
0.5	0.2	0.1	0.1	0.3	0.2	1.2	0.3	2.0	0.6904	0.6443
						2.2			0.9163	0.8673
						3.2			1.0663	1.0204
0.5	0.2	0.1	0.1	0.3	0.2	1.2	0.1	2.0	0.8518	0.8421
							0.6		0.8650	0.8322
							1.2		0.8719	0.8272
0.5	0.2	0.1	0.1	0.3	0.2	1.2	0.3	1.0	0.8811	0.5767
								2.0	0.8924	0.5841
								3.0	0.9672	0.5885

**Table 5:** Outcomes of $- f^{\prime\prime}\left( 0 \right)$ versus $M$, $A_{1}$, $A_{2}$, $Pe$ and $Lb$.

Parameters					$- \chi^{\prime}\left( 0 \right)$	
$M$	$A_{1}$	$A_{2}$	$Pe$	$Lb$	$\beta = 0.0$	$\beta = 0.3$
0.1	0.1	0.1	0.1	2	0.9749	0.9031
0.8					0.9062	0.8368
1.6					0.8459	0.7813
0.5	0.2	0.1	0.1	2	0.9334	0.8625
	0.8				0.9335	0.8624
	1.6				0.9337	0.8623
0.5	0.1	0.2	0.1	2	0.9330	0.8622
		0.8			0.9371	0.8601
		1.6			0.9286	0.8572
0.5	0.1	0.1	0.2	2	1.0098	0.9285
			1.2		1.8029	1.6069
			2.2		2.6763	2.3147
0.5	0.1	0.1	0.3	2.4	1.0393	0.9666
				2.8	1.1374	1.0636
				3.2	1.2289	1.1545

## Conclusions

The bioconvective thermal determination of Sutterby nanofluid confined via stretched cylinder has been evaluated numerically. The aspect of Darcy resistance for nonlinear radiated flow is also inspected. The numerical outcomes are listed with shooting solver which are further verified to maintain the accuracy. The major results are:

- The declining velocity change for Sutterby nanofluid is observed for Sutterby fluid parameter.
- The increment in velocity change due to Darcy resistance factor is predicted for both stretched cylinder and plate. However, the reducing change in velocity is comparatively progressive for plate.
- The temperature profile for plate and moving cylinder is lower subject to the increasing fluctuation of thermal relaxation constant.
- The stronger heat transmission is observed for sponginess parameter and Biot constant.
- The increasing concertation change of Sutterby nanofluid is noted for activation energy and thermophoresis factor.
- The increasing numerical values of local Nusselt number are predicted for thermal relaxation time constant.

## Nomenclature

$$T_{w}^{*}$$: Surface temperature
$N_{w}^{*}$: Surface microorganisms density
$C_{\infty }^{*}$: Free stream concentration
$$\left( {u_{1} ,w_{1} } \right)$$: Velocity components
$$c_{p}$$: Specific heat
$\rho_{m}$: The microorganisms density
$C$: Concentration of nanoparticles
$D^{*}_{B}$: Brownian motion coefficient
$D_{m}$: Microorganisms diffusion coefficients
$n$: Fitted rate constant,
$b$: Stand for chemotaxis constant
$\lambda_{a}$: Heat relaxation time
$N$: Density of microorganisms
$$M$$: Hartmann number
$$\beta$$: Curvature parameter
$\alpha_{1}$: Sutterby nanofluid parameter
$\alpha_{2}$: Darcy resistance parameter
$\lambda_{T}$: Thermal relaxation parameter
$$Nt$$: Thermophoresis parameter
$Rd$: Thermal radiation parameter
$\sigma$: Chemical reaction parameter
$Lb$: Bioconvection Lewis number
$\gamma$: Biot number, respectively
$Nb$: Brownian motion parameter
$\delta_{0}$: Temperature difference parameter
$j_{m}$: For local mass flux
$${\text{Re}}$$: Reynold number
$$C_{w}^{*}$$: Surface concentration
$$T_{\infty }^{*}$$: Surface temperature
$N_{\infty }^{*}$: Free stream microorganisms density
$$\rho_{f}$$: Fluid density
$$\alpha^{2}$$: Consistency index
$\rho_{p}$: Density of nanoparticles
$T$: Temperature of nanoparticles
$D^{*}_{T} \,\,$: Thermophoresis diffusion coefficient
$Kr$: Chemical reaction constant
$m^{*}$: Flow comportment index
$E$: Coefficient of activation energy
$\lambda_{b}$: Mass relaxation time
$W_{e}$: Cell swimming speed
$S^{*}$: Mixed convection parameter
$A_{1}$: Buoyancy ratio parameter
$A_{2}$: Bioconvection Rayleigh number
$$\alpha$$: Sponginess parameter
$$\Pr$$: Prandtl number
$$Le$$: Lewis number
$\theta_{w}$: Temperature ratio parameter
$E$: Activation energy parameter
$$Pe$$: Peclet number
$\varpi$: Microorganisms difference parameter
$\lambda_{C}$: Mass relaxation parameter
$q_{m}$: Local heat flux
$$j_{n}$$: Microorganisms flux
